# Avian influenza: The tip of the iceberg

**DOI:** 10.4103/1817-1737.43085

**Published:** 2008

**Authors:** Hanan Balkhy

**Affiliations:** *Department of Pediatrics, King Saud bin Abdulaziz University, King Abdulaziz Medical City, Riyadh, Saudi Arabia*

**Keywords:** Avian influenza, H5N1, review, Saudi Arabia

## Abstract

For some years now, we have been living with the fear of an impending pandemic of avian influenza (AI). Despite the recognition, in 1996, of the global threat posed by the highly pathogenic H5N1 influenza virus found in farmed geese in Guangdong Province, China, planning for the anticipated epidemic remains woefully inadequate; this is especially true in developing countries such as Saudi Arabia.

These deficiencies became obvious in 1997, with the outbreak of AI in the live animal markets in Hong Kong that led to the transmission of infection to 18 humans with close contact with diseased birds; there were six reported deaths.[1] In 2003, with the reemergence of H5N1 (considered the most likely AI virus) in the Republic of Korea and its subsequent spread to Thailand, Vietnam, Hong Kong and China. Many countries started aggressively making preparations to meet the threat.[2] The pressure for real action from governments has increased. Most developed countries have requested increased funding for the search for a more effective vaccine, for stockpiling possibly helpful antiviral drugs, and for intensifying domestic and global surveillance.[3] Most countries, however, continue to be inadequately prepared for such an epidemic, especially with regard to animal surveillance in the farm market and surveillance among migratory birds. Even now, most countries do not have the ability to detect disease among humans in the early stages of an outbreak nor do most hospitals comply with effective infection control measures that could curtail the spread of the virus in the early stages of an epidemic. In Saudi Arabia we are rapidly implementing many of these measures.[4]

## The Virus

The influenza virus is known to have an unstable character, with the ability to acquire new genetic material from other influenza viruses of similar or different serotypes, thus giving rise to new strains of influenza viruses with different genetic material. That such phenomena occur has been recognized for many years, and it is known to be responsible for the cyclical episodes of transcontinental outbreaks of influenza that have taken place. The best known is the 1918 Spanish influenza outbreak, which claimed more lives than were lost in World War I.[5] Though birds are the main reservoir, other species, including horses, pigs, and humans may easily become infected with the virus. The ability of H5N1 to infect many species rules out the possibility of eradication. Further, as the virus moves from one species to another it will exchange genetic material that will allow for easy mutations and the emergence of a virus with new characteristics.[6]

## Transmission to Humans

The most common transmission is from birds to humans. Humans exposed to infected poultry will remain in the disease incubation period from 3 to 7 days. Dead poultry seems to be the most infectious; while, handling ill poultry or defeathering, slaughtering, or cooking infected birds is believed to involve less risk. Herein lies the challenge of containing the spread of AI: in many countries back-yard farming remains a common practice and an important source of domestic income. Most disease transmission occurs from domestic animals.[7] A recent report from Azerbaijan describes transmission from wild birds, where humans acquired the infection while slaughtering wild swans;[8] however, the risk of wild birds transmitting the disease to humans is low. The risk of human-to-human transmission is also low and the reports of the appearance of disease among clusters of patients were most likely due to exposure to infected birds as a common source of infection.[9,10] Until human-to-human transmission is better understood, human bodily fluids such as respiratory secretions, urine, feces, and blood must be considered potentially infectious.

## Clinical Features and Diagnostic Methods

Health care workers (HCWs) must strive for early detection of the disease. In the presence of risk factors for human infection with H5N1, adult patients presenting with severe and rapidly progressive community-acquired pneumonia and children presenting with respiratory tract febrile illness and radiological evidence of pneumonia should be evaluated for H5N1.[11,12] It must be remembered that some patients with AI may present primarily with diarrhea.[13] Most patients presenting with AI are below the age of 40 years, the median age being 18 years. Independent risk factors for human infection with AI were described by Dinh *et al.* in Vietnamese patients and included the preparation of sick or dead poultry for consumption and having sick poultry in the household at any time within the last 7 days; in addition, the lack of an indoor water source was also identified.[14] Raising or preparing healthy poultry for consumption was not associated with AI infection.

Relevant history also includes laboratory exposure to samples collected from humans with influenza and a history of recent travel to an area where H5N1 is endemic.

Most patients infected with AI develop bone marrow suppression, which manifests as pancytopenia, particularly lymphopenia, neutropenia, and mild to moderate thrombocytopenia. In advanced cases, disseminated intravascular coagulation (DIC) and elevated D-dimer levels with evidence of hemorrhage is described. Elevated serum levels of lactate dehydrogenase and the presence of lymphopenia are associated with poorer outcome, with greater likelihood of complications like multiorgan failure, cardiac and renal dysfunction, pulmonary hemorrhage, and healthcare-related infections, especially ventilator-associated pneumonia.[15–19]

Specialized laboratories capable of PCR testing and equipped with appropriate primers for the current AI strains are important for early detection of an outbreak. Biosafety level-II laboratories which can detect infection within 4–6 h are needed.[20] Throat swabs are preferred over nasal swabs as samples to be sent for testing because of the lower viral burden in the nose. In cases where the suspicion of AI is high and initial testing is negative, it is important to send serial samples. Urine and fecal samples are of much lower diagnostic value. While rapid assays for detecting influenza antigens are available, they fail to differentiate between human and AI viruses.

Prompt reporting of the presence of suspected or confirmed cases of HP H5N1 is essential. Clinicians must be aware of how to inform appropriate hospital authorities and relevant governmental bodies in order to trigger public health defense mechanisms at the earliest.

## Treatment

The fatality rate due to infections with HP H5N1 is as high as 62%; fortunately, only 373 cases have been identified as of March 2008.[21] There remains insufficient data on the best care for such patients. There is no standard approach towards management and any guidance available is based on anecdotal experiences.[22] There is evidence that early treatment of definitively documented AI is associated with better outcomes and increased survival rates as compared to late interventions.[23] Early detection, therefore, can be lifesaving.

The options for pharmacotherapy remain limited; the choice is between neuraminidase inhibitors, of which most experience has been with oseltamivir, and the adamantanes, which are inhibitors of the ion channel activity of the M2 membrane protein of influenza A viruses, such as amantadine. Currently, oseltamivir remains the drug of choice because of evidence of reduced mortality when oseltamivir is initiated in the early stages of the disease. In the mouse model, a survival benefit has been demonstrated with a combination therapy of oseltamivir and amantadine over monotherapy.[24] Therefore, in areas with amantadine-sensitive H5N1, it seems reasonable to consider combination therapy in patients with progressive disease.[25]

The emergence of resistant AI virus has been detected when the virus has the N294S resistance gene which encodes for a lower susceptibility to oseltamivir. AI viruses with this specific mutation have been identified in infected human cases in Egypt, Turkey, and Indonesia. For patients who are infected with this virus, it is important to be aware of the option of using oseltamivir at a higher dose and for a longer duration (e.g., 150 mg twice daily for 10 days for adults) in those who present late or who fail to improve while on the standard dose for the drug (i.e., 75 mg twice daily for 5 days for adults).[11] Resistance to amantadine is also being reported with increasing frequency.[26]

In addition to antiviral drugs, supportive care is critical and includes fluid management, correction of hypoxemia, and prevention and management of healthcare-associated infections. The use of corticosteroids is not routine practice but it may be used in septic shock or in renal insufficiency.[22]

## Prophylaxis

The WHO guidelines recommend prophylaxis for those who have been exposed to HP H5N1 AI. Mathematical models simulating emerging outbreaks in rural Asia predict that an influenza pandemic can be prevented through the provision of targeted, mass chemoprophylaxis, along with implementation of isolation policies.[27] However, we should keep in mind that *casual use* of the very few proven antiviral agents that are available, whether as part of a planned mass prophylaxis program or as recommendations by individual physicians, may aid the development of drug resistance. Either way, these agents will become useless during a pandemic. This definitely poses tough decisions for health care policymakers.[28]

## Immunization

As with all viruses of rapidly changing antigenicity, the hope for developing an effective AI vaccine is a major challenge.[29] One part of the WHO Global Influenza Program is the development of representative H5N1 candidate vaccine viruses. Since it is not known *which* specific H5N1 virus will become the pandemic virus, efforts are in place to prepare for the most likely viruses, including *both* clade 1 and clade 2 influenza viruses.[30]

## H5N1 Outbreak in the Kingdom of Saudi Arabia

**Figure 1:**
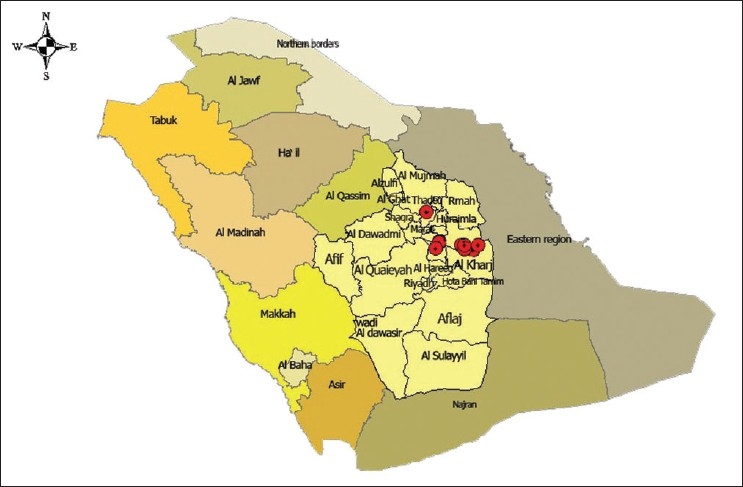
Location of poultry farms affected with H5N1 AI in Saudi Arabia

The first cases of AI in the Kingdom were reported from a farm in Al Kharj District on April 2, 2007, when 1500 birds died. This occurrence was reported to the Saudi Ministry of Agriculture (MOA)[31] [Figure 1]. There was an intensive public health infection control response to this event. PCR testing on the dead birds quickly confirmed the presence of HP H5N1. The remainder of the flock (50,000 birds) was promptly culled.

**Figure 2:**
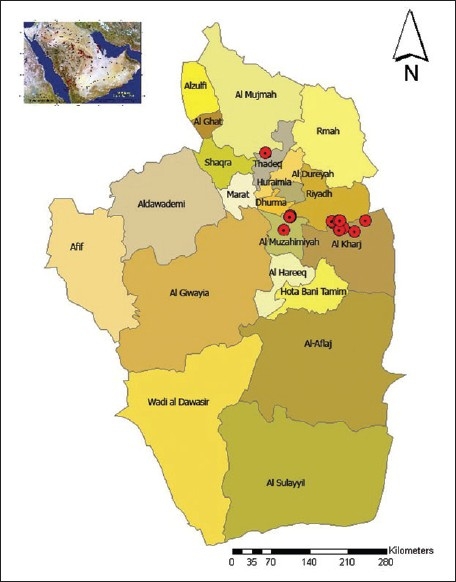
Location of poultry farms affected with H5N1 AI in the Riyadh district area

The farm was disinfected and designated as an ‘infected area.’ Human contacts were closely followed for possible acquisition of disease. Despite these measures, the outbreak spread to the nearby poultry farms in Drumaa, Almazahmiah, Alhayathem, and several others [Figure 2].[32] It is speculated that the birds were initially infected by migratory wild birds.[33]

Efforts to control such outbreaks require intense collaboration between many governmental and non-governmental agencies. In the Kingdom of Saudi Arabia the consequences of an outbreak during the hajj will have a global impact as the Kingdom hosts over 2.5 million pilgrims a year from all over the world.[4,34] The MOA has detailed the emergency measures and a prevention plan for HP AI in the Kingdom of Saudi Arabia. These measures include several critical steps to prevent the introduction of HP H5N1 into poultry farms: e.g., banning the importation of poultry and poultry products and food from countries with endemic AI; isolating those who return from endemic areas to work on poultry farms for at least 10 days; isolating poultry farms and storage areas from exposure to migratory and wild birds; and, lastly but importantly, educating workers and owners of poultry farms on appropriate preventive measures and early reporting of ill or dying poultry to the MOA.[35] Strict policies on destruction of infected and exposed birds, as well as farm decontamination, are clearly detailed in the plan. In addition, the designation of an area within a 5-km radius of an infected farm as a ‘restricted area’ is a critical factor in successful disease control; this is necessary in order to survey the area for newly infected birds. Within the restricted area, all birds dying due to any reason are to be dealt with as if they have died from AI. Additional measures include banning the exit of any heavy machinery or birds from the restricted area for a period of 6 months from the date of identification of the last diseased bird. Only after 6 months of consistently documented absence of newly identified cases can the farm and the surrounding area be opened up for full access.[35]

At the Ministry of Health (MOH) level, many educational programs are now in place to educate HCWs on the importance of standard and extended precautions, isolation policies and procedures, and hand hygiene. Such efforts are vital in this phase of the pandemic, when no human cases have yet been identified. Even though these measures will be helpful, successful compliance and adherence to these policies calls for a change in HCWs' behavior and this has its own unique barriers to implementation, a discussion of which is beyond the scope of this review.

The MOH has identified a number of protocols to be followed in the event of human cases being suspected or identified. These include standard operating procedures for individuals under investigation for HP H5N1, case definitions, data collection forms, emergency contact numbers, public health measures, and educational material for HCWs and the public. All efforts have been in collaboration with the concerned governmental bodies in order to standardize these approaches.

Fortunately, during the outbreak in the Kingdom of Saudi Arabia, none of the human contacts of infected birds were identified as having AI. As we remain in the early phases of the pandemic, we should bear in mind that once the virus acquires the genetic material that allows for more efficient human-to-human transmission the number human infections will rise and we can expect an explosive pandemic.

## Conclusion

Even as we hope that AI will remain a serious problem involving only birds, it is crucial to prepare for a possible major outbreak of human disease, especially since we have historical evidence that influenza pandemics do recur, typically several times each century.[36] This observation has catalyzed the development of global preparedness plans by the WHO in which all nations become integral parts. All governments would be well advised to develop and be ready to execute emergency plans in the event of what appears to be an inevitable pandemic.[37,38]
